# Plasma High-Mannose and Complex/Hybrid N-Glycans Are Associated with Hypercholesterolemia in Humans and Rabbits

**DOI:** 10.1371/journal.pone.0146982

**Published:** 2016-03-21

**Authors:** Liang Bai, Qianwei Li, Lingmei Li, Yan Lin, Sihai Zhao, Weirong Wang, Rong Wang, Yongqin Li, Jiangbei Yuan, Chengjian Wang, Zhongfu Wang, Jianglin Fan, Enqi Liu

**Affiliations:** 1 Research Institute of Atherosclerotic Disease, Xi’an Jiaotong University Cardiovascular Research Center, Xi’an, Shaanxi, 710061, China; 2 Laboratory Animal Center, Xi’an Jiaotong University Health Science Center, Xi’an, Shaanxi, 710061, China; 3 Educational Ministry Key Laboratory of Resource Biology and Biotechnology in Western China, College of Life Science, Northwest University, Xi’an, 710069, China; 4 Department of Cardiology, the Second Affiliated Hospital of Xi’an Jiaotong University, Xi’an, Shaanxi, 710004, China; 5 Department of Molecular Pathology, Interdisciplinary Graduate School of Medicine and Engineering, University of Yamanashi, Yamanashi, 409–3898, Japan; Showa University School of Pharmacy, JAPAN

## Abstract

N-glycans play important roles in various pathophysiological processes and can be used as clinical diagnosis markers. However, plasma N-glycans change and their pathophysiological significance in the setting of hypercholesterolemia, a major risk factor for atherosclerosis, is unknown. Here, we collected plasma from both hypercholesterolemic patients and cholesterol-fed hypercholesterolemic rabbits, and determined the changes in the whole-plasma N-glycan profile by electrospray ionization mass spectrometry. We found that both the hypercholesterolemic patients and rabbits showed a dramatic change in their plasma glycan profile. Compared with healthy subjects, the hypercholesterolemic patients exhibited higher plasma levels of a cluster of high-mannose and complex/hybrid N-glycans (mainly including undecorated or sialylated glycans), whereas only a few fucosylated or fucosylated and sialylated N-glycans were increased. Additionally, cholesterol-fed hypercholesterolemic rabbits also displayed increased plasma levels of high-mannose in addition to high complex/hybrid N-glycan levels. The whole-plasma glycan profiles revealed that the plasma N-glycan levels were correlated with the plasma cholesterol levels, implying that N-glycans may be a target for treatment of hypercholesterolemia.

## Introduction

N-glycans constitute a basic component of cell membrane and secreted proteins and play important roles in many physiological and pathological processes[1–3]. N-glycans are covalently attached to proteins at asparagine residues by an N-glycosidic bond[4]. In general, physiological functions of N-glycans can be classified into two categories: (1) the structural and modulatory properties and (2) the specific recognition of N-glycans by other molecules[5, 6]. Furthermore, N-glycans are involved in the pathogenesis of human diseases[7]. For example, defect of N-glycan synthesis leads to a variety of human diseases[8] and abnormality in N-glycans participates in cancer metastasis and invasion[9–11].

Most plasma proteins are covalently associated with N-glycans because N-glycans mediate many functions of glycoproteins such as their conformation, folding, solubility and antigenicity as well as cell-matrix and cell-cell interactions[6]. Because of these vital roles in so many biological processes, plasma N-glycans can be used as a diagnostic marker for the diagnosis and monitoring of various chronic diseases[12–14] and cancers[15]. In blood circulation, the lipids are transported by proteins called lipoproteins. These lipoproteins can be classified into different categories based on their size, density, migration ability on the electrophoresis and their biological functions. In general, very low density lipoproteins (VLDL), intermediate density lipoproteins (IDL), low density lipoproteins (LDL), lipoprotein(a) [Lp(a)] and remnant lipoproteins (also called apoB-containing particles) are considered “atherogenic” while high density lipoproteins (HDL) (also called apoAI-containing particles) are “anti-atherogenic”[16]. In hypercholesterolemic patients, plasma levels of apoB-containing lipoproteins as mentioned above are markedly increased therefore these patients are susceptible to the development of atherosclerosis[17]. It has been reported that there are strong and consistent associations between certain glycans and lipids in human[18]. However, in the setting of hypercholesterolemia, it is unknown whether plasma N-glycans are also changed and their pathophysiological significance remains elusive. In this respect, we envisioned that plasma N-glycans may be affected by the presence of high levels of atherogenic lipoproteins. In this study, we analyzed the whole-plasma N-glycan profiles of both hypercholesterolemic patients and rabbits using electrospray ionization mass spectrometry (ESI-MS). Our data showed that hypercholesterolemia is associated with the increased N-glycan levels in plasma, including high-mannose and complex/hybrid (undecorated/sialylated). These results provide the first evidence that N-glycan levels are elevated in the setting of hypercholesterolemia and such changes open a new path for the diagnosis and treatment of both hypercholesterolemia and atherosclerosis in future.

## Materials and Methods

### Human subjects

We recruited 9 male hypercholesterolemic patients and 9 male healthy volunteers at the Second Affiliated Hospital of Xi’an Jiaotong University, Xi’an, China. The clinical information is shown in Table 1. The blood samples were taken after fasting overnight. All protocols were approved by the ethics committee of the Second Affiliated Hospital of Xi’an Jiaotong University and informed written consents were obtained from all subjects. The investigation was conformed to the principles outlined in the Declaration of Helsinki for the use of human subjects.

**Table 1:** Information of healthy subjects and hypercholesterolemic patients.

Groups	Healthy subjects (n = 9)	Hypercholesterolemic patients (n = 9)
**Age (years)**	51±2	54±2
**TC (mg/dL)**	164±4	290±28***
**TG (mg/dL)**	95±9	153±22*
**LDL-C (mg/dL)**	103±2	249±32***
**HDL-C (mg/dL)**	27±3	32±3
**Glucose (mg/dL)**	84±4	87±3

Data are expressed as the mean ± SEM. n = 9 for each group.

*P<0.05,

***P<0.001 (Student’s *t* test).

### Animals

Japanese white rabbits (male, 4-mon, n = 5) were provided by the Institute of Biological Products, Wuhan, China. All rabbits used in this study were housed in a clean animal facility with a 12h light/12h dark cycle. Rabbits were fed with a chow diet first and then changed to a high cholesterol diet containing 0.3% cholesterol and 3% bean oil (Vital River Laboratories, Beijing, China) for 14 weeks. Rabbits were given a restricted amount of each diet (100 g/rabbit per day) with free access to drinking water. All animal experiments were approved by the Xi’an Jiaotong University Institutional Animal Care and Use Committee, and performed according to the Guidelines for Animal Experimentation of Xi’an Jiaotong University and the Guide for the Care and Use of Laboratory Animals Published by the US National Institutes of Health.

### Biochemical assays

The blood samples obtained from either humans or rabbits were collected into a tube containing EDTA. The plasma was separated after centrifuged at 3,000 rpm/min for 10 min. Total cholesterol (TC), triglycerides (TG), low density lipoprotein-cholesterol (LDL-C), high density lipoprotein-cholesterol (HDL-C) and glucose (Biosino Bio-technology and Science Inc., Beijing, China) contents were determined as described previously[19]. For analysis of apoB-containing lipoproteins including VLDL, IDL, and LDL subfractions, the plasma lipoprotein profile was analyzed using gradient gel electrophoresis (Lipoprint LDL Subfraction System, Quantimetrix Corporation, Redondo Beach, CA, USA) according to the manufacturer’s instructions[20]. The subfractions were then quantified using NIH image program version 1.62 (Bethesda, MD, USA).

### Pretreatment of plasma samples

The plasma was collected and dialyzed against Milli-Q water at 4°C for 72 hours. The water was changed every 12 hours. The dialyzed plasma samples were finally lyophilized and stored at −20°C until use[21].

### Enzymatic release and purification of N-glycans

The lyophilized plasma sample (3 mg) was dissolved in 300 μl of protein denaturation solution, containing 0.4 M DL-Dithiothreitol and 5% sodium dodecyl sulfate, and denatured at 100°C for 10 min. After the samples became cooled at room temperature, 30 μl of 1 M sodium phosphate buffer (pH 7.5), 30 μl of 10% aqueous NonidetP-40 (v/v), and 1 μl of Peptide -N-Glycosidase F solution (500 units) (New England BioLabs, Ipswich, MA, USA) were added and then incubated in 37°C water bath for 24 h, which was stopped by boiling at 100°C for 5 min. To remove protein, the samples were loaded onto a SepPak C18 solid phase extraction column (Waters, Milford, MA, USA) and the N-glycan fractions were desalted using a graphitized carbon SPE column (Alltech Associates, Deerfield, IL, USA). The target N-glycans were eluted with 25% acetonitrile (Fisher Scientific, Fairlawn, NJ, USA) containing 0.01% trifluoroacetic acid. The eluates were dried under a stream of nitrogen gas and stocked until use[22, 23].

### ESI-MS

N-glycan analysis was performed with an LTQ XL linear ion trap electrospray ionization mass spectrometer (Thermo Scientific, Waltham, MA, USA). Briefly, N-glycan samples were directly infused via a Rheodyne loop injector with a volume of 2 μl and subsequently brought into the electrospray ion source by a stream of 50% methanol (v/v) at a flow rate of 200 μl/min. The molecular ions formed in the ion source were inspired and transferred to the ion trap via capillary and quadrupole. Therefore, molecular ions were detected. The spray voltage was set at 4 kV, with a sheath gas (nitrogen gas) flow rate of 30 arb., an auxiliary gas (nitrogen gas) flow rate of 5.0 arb., a capillary voltage of 37 V, a tube lens voltage of 250 V, and a capillary temperature of 37°C. For MS/MS analysis, N-glycans were subjected to fragmentation by collision induced decomposition, with helium as the collision gas. Collision parameters were left at default values with a normalized collision energy degree of 60 and an isotope width of m/z 3.00. Activation Q was set at 0.25, and activation time, at 30 ms. The MS and MS/MS data were recorded using LTQ Tune software (Thermo Scientific, Waltham, MA, USA) [22–24]. Glycan compositions and sequences were assigned manually. Then, we validated glycan structures either with reference to previous literature reports or by checking with GlycoWorkbench in databases such as CFG, CarbBank, and GLYCOSCIENCES.

### Statistical Analysis

Results are presented as mean ± SEM. Statistical analysis was performed by two-tailed Student’s *t* test using GraphPad Prism 5 software (GraphPad Software Inc., San Diego, CA, USA). A *P* value less than 0.05 was considered statistically significant.

## Results

### Plasma lipids in hypercholesterolemic patients

**Fig 1:**
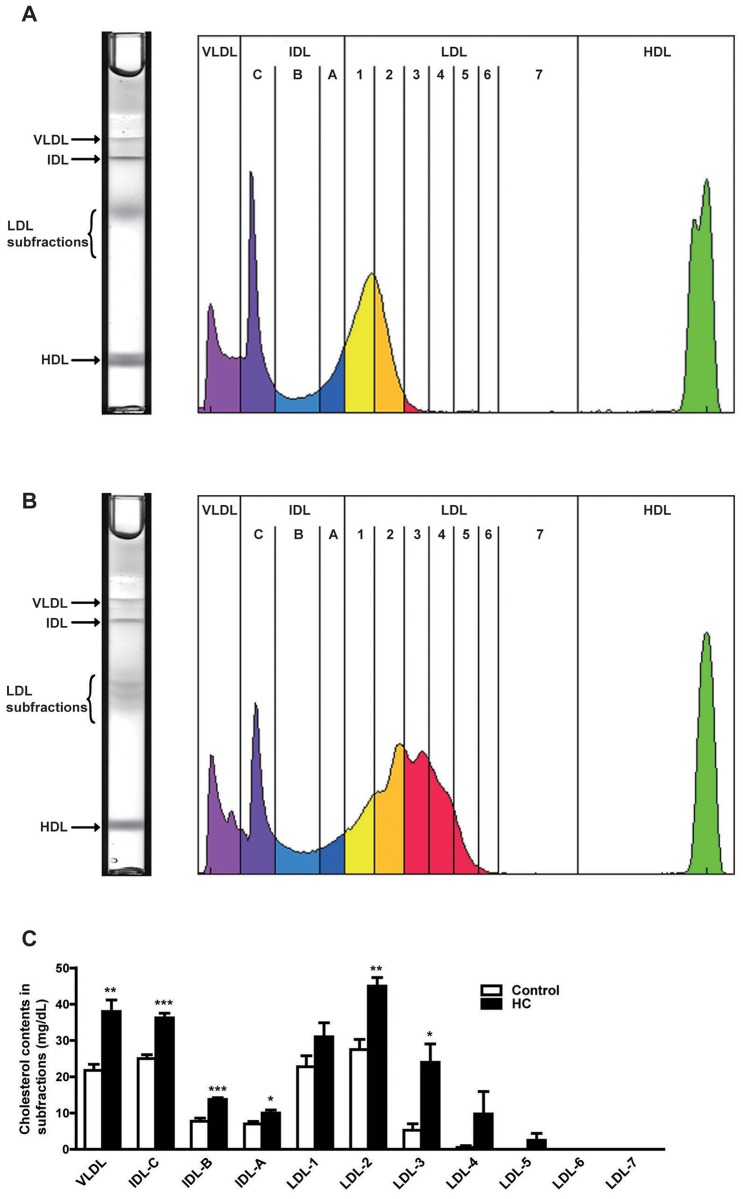
ApoB-containing particle subfractions in plasma of hypercholesterolemic patients and healthy subjects. Plasma lipoproteins were resolved to discrete bands consisting of very low density lipoproteins (VLDL), intermediate density lipoproteins (IDL) bands C, B, A, low density lipoproteins (LDL) subfractions 1 to 7, and an high density lipoproteins (HDL) band using a lipoprint system. Representative image of LDL in polyacrylamide tube gel electrophoresis and the corresponding computerized lipoprotein profile in plasma of healthy subjects (A) and hypercholesterolemic patients (B). ApoB-containing particle subfractions were quantified in plasma of hypercholesterolemic patients and healthy subjects (C). Data are expressed as the mean ± SEM. n = 4 for each group. *P<0.05, **P<0.01, ***P<0.001 vs. control. HC, hypercholesterolemia.

Compared with healthy subjects, the hypercholesterolemic patients exhibited dramatically increased plasma lipid levels, including a 1.8-fold increase of TC, 1.6-fold increase of TG, and 2.4-fold increase of LDL-C, while no changes of HDL-C and glucose were observed (Table 1). Analysis of plasma lipoprotein profiles revealed that all apoB-containing particles (VLDLs, IDLs, and LDLs) were markedly increased in hypercholesterolemic patients compared with the healthy controls (Fig 1A–1C). Furthermore, LDL subfractions, especially those with smaller size (fractions 3, 4 and 5) remarkably appeared in the hypercholesterolemic patients.

### Plasma N-glycan profiling in the hypercholesterolemic patients

**Fig 2:**
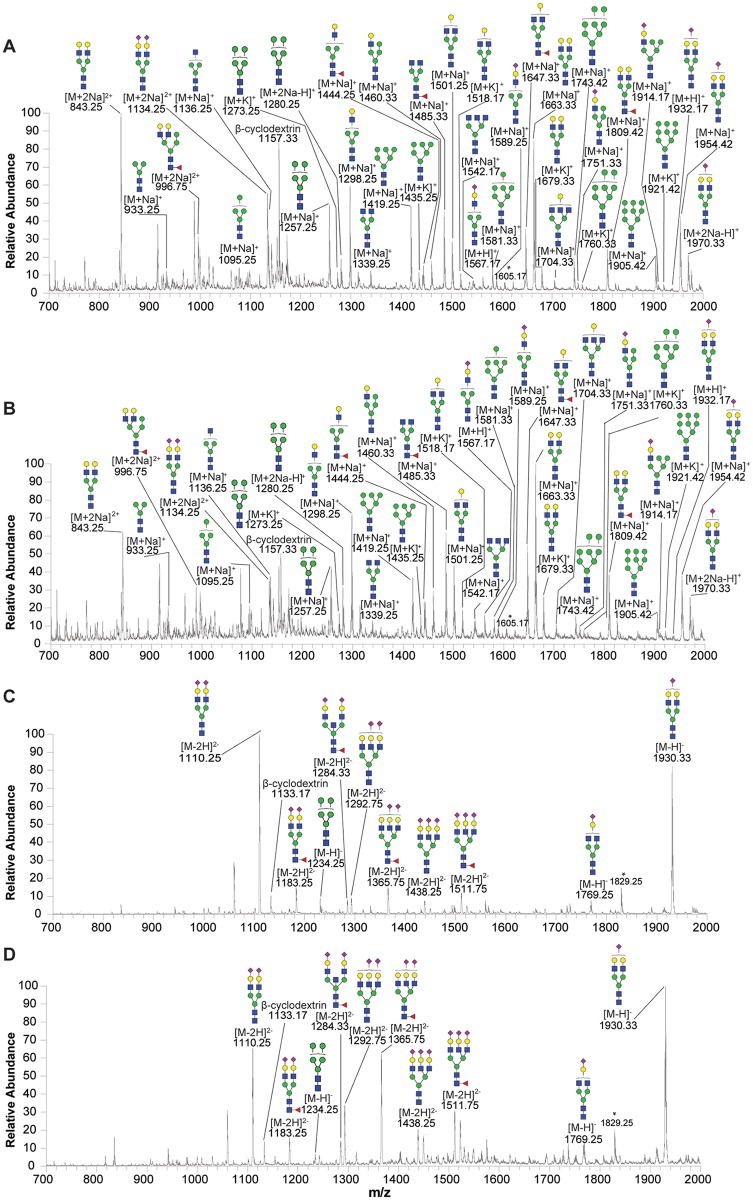
ESI-MS profiles of the plasma N-glycans released from humans. Representative N-glycan profiles of the whole plasma from either healthy (A and C) or hypercholesterolemic subjects (B and D). A and B show mass spectra of N-glycans in positive-ion mode; and C and D show mass spectra in negative-ion mode. Putative glycan compositions were presented on the basis of the MS/MS analysis. Structural formulas: blue square, N-acetylglucosamine; green circle, mannose; yellow circle, galactose; red triangle, fucose; purple diamond, N-acetylneuraminic acid.

**Fig 3:**
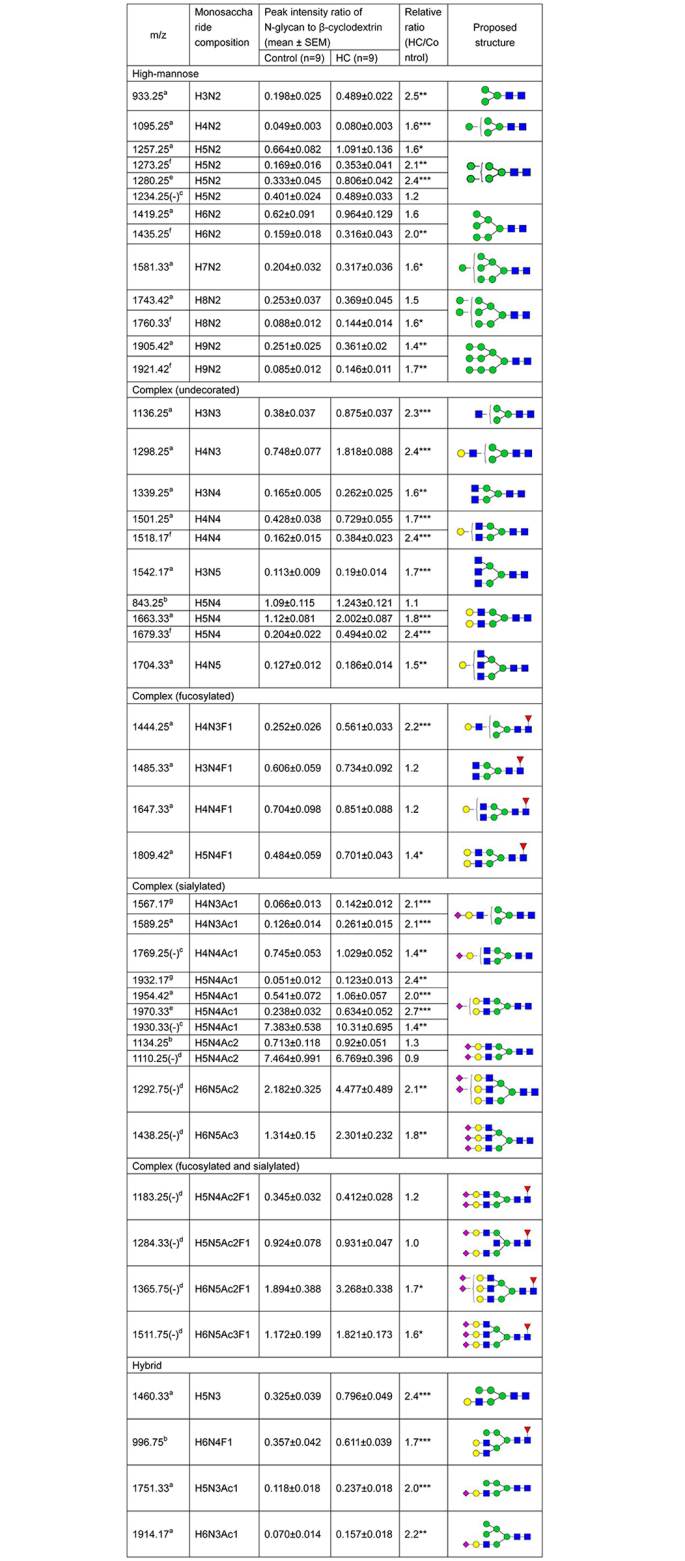
Plasma N-glycans compositions and proposed structures of healthy subjects and hypercholesterolemic patients. Remarks: (-), the N-glycans in negative-ion mode; a-[M+Na]^+^; b-[M+2Na]^2+^; c-[M-H]^-^; d-[M-2H]^2-^; e-[M+2Na-H]^+^; f-[M+K]^+^; g-[M+H]; HC, hypercholesterolemia; H, hexose; N, N-acetylhexosamine; Ac, N-acetylneuraminic acid; F, fucose; Gc, N-glycolylneuraminic acid; *P<0.05 **P<0.01, ***P<0.001. Structural formulas: blue square, N-acetylglucosamine; green circle, mannose; yellow circle, galactose; red triangle, fucose; purple diamond, N-acetylneuraminic acid.

As shown in Fig 2, ESI-MS analysis detected mass spectra of plasma N-glycans under either positive or negative-ion mode of both healthy subjects and hypercholesterolemia patients. In total, ESI-MS analysis revealed 48 peaks (37 peaks under positive-ion mode and 11 peaks under negative-ion mode) representing 32 N-glycans in both healthy controls and hypercholesterolemic subjects: (Figs 2 and 3). Tandem MS of the four representative glycans (mass over charge values at 1743.42, 1501.25, 1485.33 and 1930.33) were used to confirm the N-glycan structures (S1 Fig). Although 32 N-glycans were present in both healthy subjects and hypercholesterolemic patients, there was a difference in contents of these glycans between two groups. We found that 27 kinds of N-glycans were significantly higher in plasma of hypercholesterolemic patients than those of the control group (Fig 3). Heat map showed that three groups of N-glycans were apparently increased in the hypercholesterolemic patients (S2A Fig): they were high-mannose with mass over charge values at 933.25, 1095.25, 1257.25, 1273.25, 1280.25, 1419.25,1435.25, 1581.33, 1743.42, 1760.33, 1905.42 and 1921.42 *m/z* (S2B Fig), complex (undecorated) N-glycans at 1136.25, 1298.25, 1339.25, 1501.25, 1518.17, 1542.17, 1663.33, 1679.33 and 1704.33 *m/z* (S2C Fig) and 2 complex (fucosylated) N-glycans at 1444.25 and 1809.42 *m/z* (S2D Fig). Complex (fucosylated and sialylated) at 1365.75 (-) and 1511.75 (-) *m/z* (S2E Fig), and complex (sialylated) at 1567.17, 1589.25, 1769.25 (-), 1932.17, 1954.42, 1970.33, 1930.33 (-), 1292.75 (-) and 1438.25 (-) *m/z* were also higher in the hypercholesterolemic patients than that of controls (S2F Fig). In addition, the level of hybrid N-glycans at 1460.33, 996.75, 1751.33 and 1914.17 *m/z* dramatically increased in the hypercholesterolemic patients compared with that of controls (S2G Fig). Pearson correlation coefficient indicated that 16 N-glycan levels were significantly correlated with cholesterol levels (0.48≤r≤0.69, *P*<0.05).

### Plasma lipids in the cholesterol-fed rabbits

**Fig 4:**
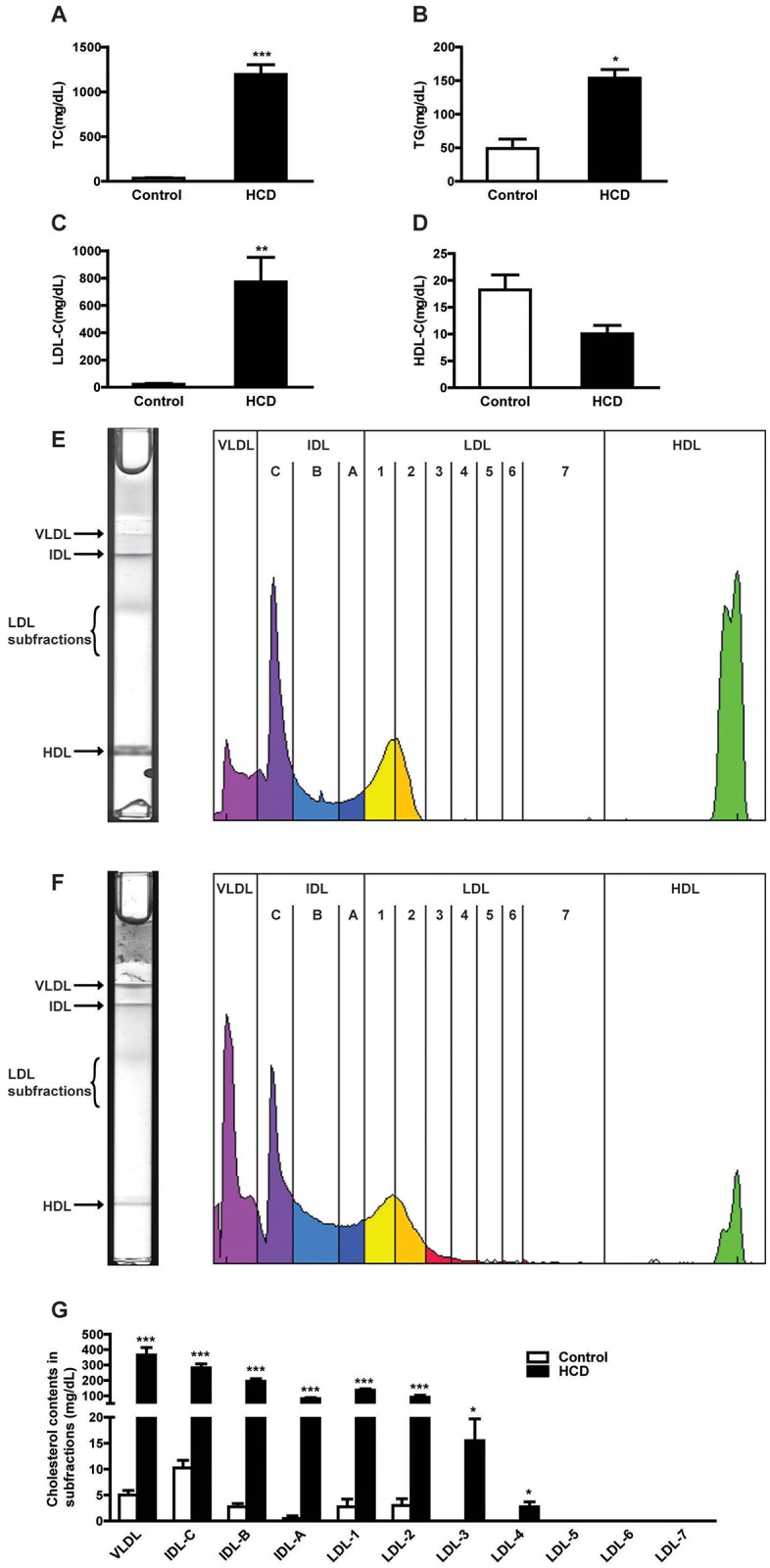
Plasma lipids and lipoprotein profiles of chow diet and high cholesterol-fed rabbits. Total cholesterol (TC) (A), triglyceride (TG) (B), low density lipoprotein-cholesterol (LDL-C) (C), and high density lipoprotein-cholesterol HDL-C (D) were measured. Representative image of LDL in polyacrylamide tube gel electrophoresis and the corresponding scanned and computerized lipoprotein profile in plasma of controls (E) and hypercholesterolemic rabbits (F). ApoB-containing particle subfractions were quantified in plasma of controls and hypercholesterolemic rabbits (G). Data are expressed as the mean ± SEM. n = 4 or 5 for each group. *P<0.05 **P<0.01, ***P<0.001. HCD, high cholesterol diet.

To determine whether the plasma N-glycan compositions can be induced experimentally, we compared plasma derived from cholesterol-fed rabbits with that from normal rabbits. Cholesterol-feeding dramatically increased plasma lipid levels in rabbits: 33-fold increase of TC, 3-fold increase of TG, 35-fold increase of LDL-C whereas HDL-C levels were decreased compared to the control rabbits (Fig 4A–4D). Analysis of lipoprotein profiles revealed that all apoB-containing particles were markedly increased in cholesterol-fed rabbits (Fig 4E–4G). Similar to human hypercholesterolemia, small-sized LDLs (designated as fractions 3–4) became prominent whereas they were not present in normal rabbits (Fig 4E–4G).

### Plasma N-glycan profiling in hypercholesterolemic rabbits

**Fig 5:**
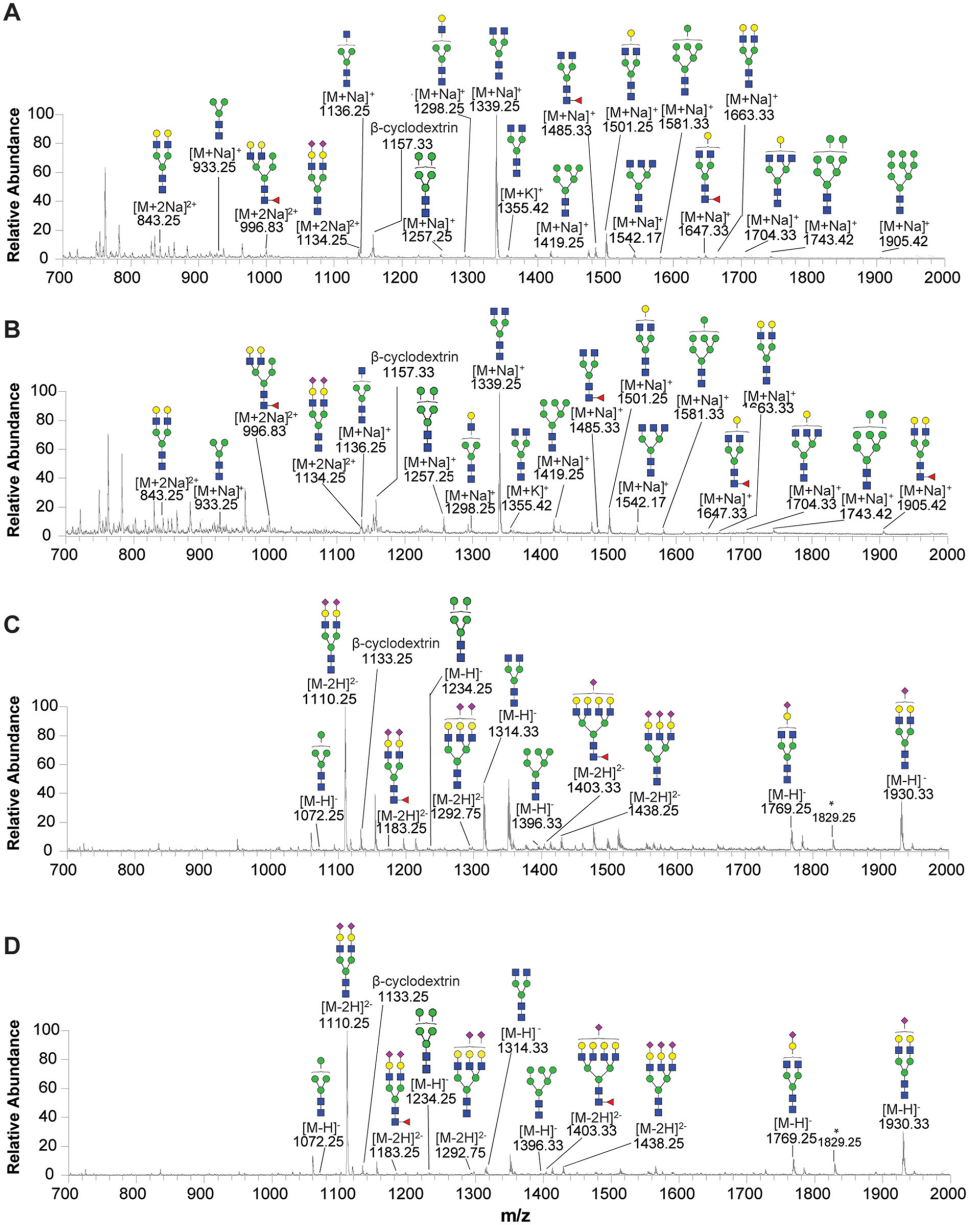
ESI-MS profiles of the plasma N-glycans released from rabbits. Representative N-glycan profiles of the whole plasma from either normal (A and C) or hypercholesterolemic rabbits (B and D). A and B show mass spectra of N-glycans in positive-ion mode; and C and D show mass spectra in negative-ion mode. Structural formulas: blue square, N-acetylglucosamine; green circle, mannose; yellow circle, galactose; red triangle, fucose; purple diamond, N-acetylneuraminic acid.

In total, ESI-MS analysis revealed 31 peaks (19 peaks under positive-ion mode and 12 peaks under negative-ion mode) representing 24 N-glycans in rabbit plasma, which included 7 high-manose, 16 complex N-glycans and 1 hybird N-glycans (Fig 5). Tandem MS of the four representative glycans were used to confirm the glycan compositions and sequences (S3 Fig). In rabbits plasma, high-manose accounted for 29% (7/24), complex N-glycans accounted for 67% (16/24) and hybird N-glycans accounted for 4% (1/24), whereas 22% (7/32) of high-manose, 66% (21/32) of complex and 12% (4/32) of hybird N-glycans in human plasma. Though the percentage of complex N-glycans were similar in human and rabbit plasma, but the component were different. In rabbits, there were 44% (7/16) of the undecorated and 56% (9/16) of the sialylated or fucosylated complex N-glycans, whereas 33% (7/21) of undecorated and 67% (14/21) of the decorated N-glycans in human.

**Fig 6:**
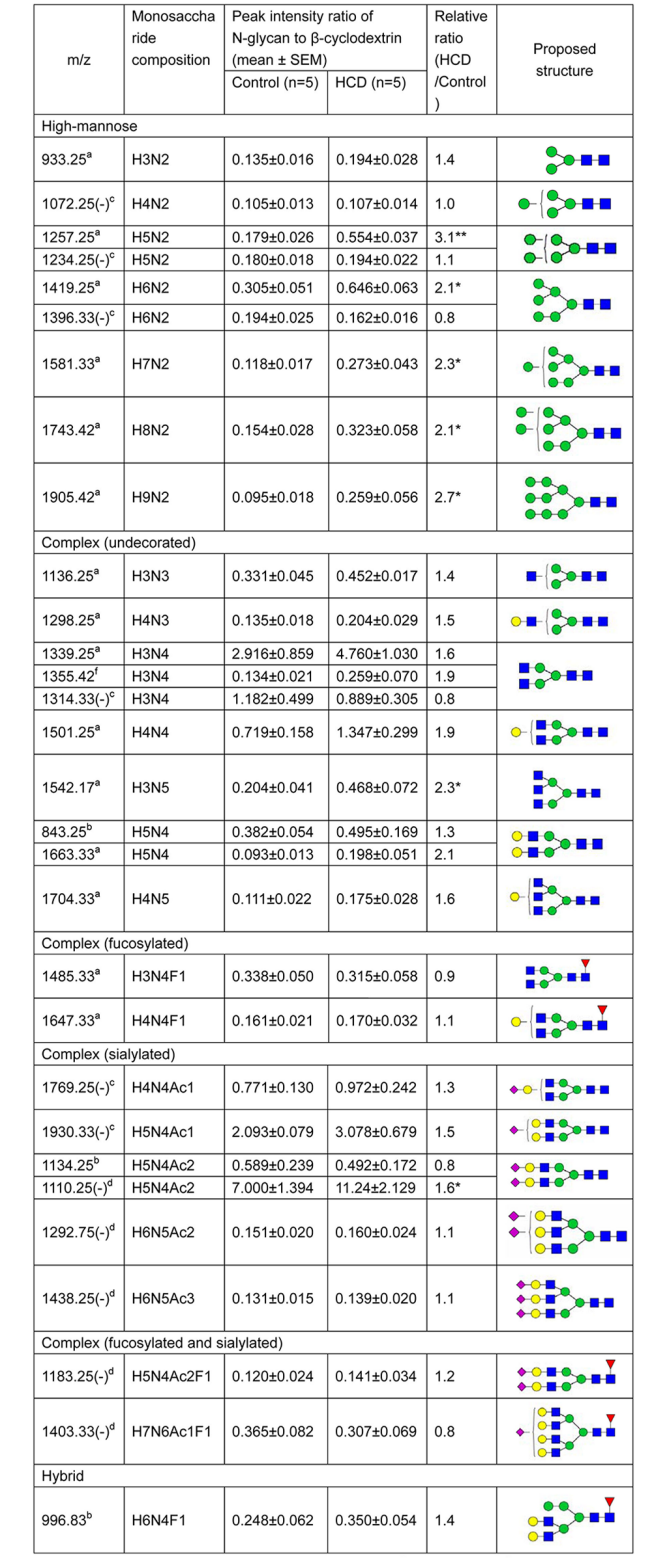
Plasma N-glycans compositions and proposed structures of controls and hypercholesterolemic rabbits. Remarks: (-), the N-glycans in negative-ion mode; a-[M+Na]^+^; b-[M+2Na]^2+^; c-[M-H]^-^; d-[M-2H]^2-^; e-[M+2Na-H]^+^; f-[M+K]^+^; g-[M+H]; HCD, high cholesterol diet; H, hexose; N, N-acetylhexosamine; Ac, N-acetylneuraminic acid; F, fucose; Gc, N-glycolylneuraminic acid; *P<0.05 **P<0.01. Structural formulas: blue square, N-acetylglucosamine; green circle, mannose; yellow circle, galactose; red triangle, fucose; purple diamond, N-acetylneuraminic acid.

Furthermore, we found that 13 kinds of N-glycans in the hypercholesterolemic rabbits were higher (≥1.5-fold increase and 54% of them were statistically significant) than those in normal rabbits (Fig 6). Similar to the results obtained from human, most of these N-glycans belonged to high-mannose or complex (undecorated) (S4A Fig). High-mannoses at 1257.25, 1419.25, 1581.33, 1743.42 and 1905.42 *m/z* (S4B Fig), complex (sialylated) N-glycans at 1930.33 (-) and 1110.25 (-) *m/z* (S4C Fig), and complex (undecorated) N-glycans at 1298.25, 1339.25, 1355.42, 1501.25, 1542.17, 1663.33 and 1704.33 *m/z* were higher in the plasma of hypercholesterolemic rabbits than that in the controls (S4D Fig). Additionally, 8 N-glycan levels were dramatically correlated with cholesterol levels (0.65≤r≤0.85, *P*<0.05).

## Discussion

Hypercholesterolemia is a major risk factor for the development of atherosclerosis; therefore, to find a new diagnostic maker is essential for the prevention and the treatment for cardiovascular disease[25–27]. Global N-glycan profiling of human plasma based on mass spectrometry techniques has provided an alternative means to explore potentially promising markers for human diseases[28]. In the current study, we characterized the whole-plasma N-glycan profiling of both hypercholesterolemic patients and rabbits, and demonstrated for the first time that plasma levels of high-mannose and complex/hybrid N-glycans are associated with plasma cholesterol levels.

In human hypercholesterolemia, the major atherogenic lipoproteins are LDLs whereas in cholesterol-fed rabbits, the major atherogenic lipoproteins are those of intestinally and hepatically derived remnant lipoproteins called β-VLDLs. Although lipoprotein are quite different in compositions because of different pathogenesis between hypercholesterolemic patients and experimental rabbits, high plasma N-glycan levels are correlated with both human and rabbit plasma cholesterol levels, suggesting that N-glycans are simultaneously changed under the hypercholesterolemic state regardless of lipoprotein profiles (LDL vs β-VLDL). However, 32 N-glycans were detected in human plasma whereas only 24 N-glycans were observed in rabbit plasma, suggesting that the profiles of plasma lipoprotein may interacts with N-glycan compositions or there is a species difference between human and rabbit. In human plasma, the decorated complex and hybrid N-glycans accounted for main component, whereas high-mannose and undecorated complex N-glycans were major component in rabbits plasma, implying the different complexity of glycome in various species. Interestingly, either in hypercholesterolemic patients or high-cholesterol fed rabbits, the levels of N-glycan were higher than that of normal group. Pearson correlation coefficient showed that N-glycan levels were significantly positively correlated with cholesterol levels both in human (0.48≤r≤0.69) and rabbits (0.65≤r≤0.85) (*P*<0.05). However, it is currently unknown that how hypercholesterolemia affects N-glycans or how these two interacts with each other. It also remains unclear what the pathophysiological significance of plasma N-glycans is. Do N-glycans play any roles in the pathogenesis of atherosclerosis? Or can we use these N-glycans as a marker for diagnosis of atherosclerotic disease?

Many plasma glycoproteins with N-glycosylation change are implicated in serious pathological conditions such as cancers, cardiovascular diseases and autoimmune diseases[29–34]. Their glycosylation pattern could be used as disease biomarkers for early diagnosis or anchor points for targeted treatments. Fox example, the fucosylation and sialylation of Alpha-1-acid glycoprotein are significantly increased in rheumatoid arthritis[34, 35]. Changes in the glycosylation of Alpha-2-macroglobulin and Alpha-2-HS-glycoprotein have been associated with cancer and autoimmune diseases[34]. The N-glycosylation of lectin-like oxidized low density lipoprotein receptor-1 has been shown to modulate the pathogenesis of atherosclerosis[29, 31]. N-glycosylation defect on scavenger receptor expressed by endothelial cells(SREC-I) could impair the uptake of modified LDL into macrophages and endothelial cells[32]. Treatment with tumor necrosis factor α (TNFα) in human umbilical vein endothelial cells (HUVECs) was shown to increase the surface expression of high mannose/hybrid N-glycans [36]. The increased high mannose/hybrid N-glycans acted as ligands further contribute to monocytes rolling and adhesion to endothelial cells, which process the development of atherosclerosis[36]. Macrophage-expressed mannose-binding lectin (MBL) was also found to be enriched in early stage of atherosclerotic lesions[37]. When exposed to oscillatory shear or under TNFα stimulation, mannose-specific lectin staining intensity was increased in human aortic endothelial cells and at sites of early atherosclerotic lesions in both mice and human arteries[38]. Conversely, enzymatic removal of high-mannose N-glycans, or masking mannose residues with lectins, strongly decreased monocyte adhesion under the flow[38]. Therefore, N-glycans profile is analogous to a molecular zip code that regulates leukocyte trafficking[39].

apoB-100 plays an important role in the assembly of VLDL and lipoproteins, and transports the majority of plasma cholesterol. Its N-glycosylation sites are occupied by high mannose, diantennary complex, and hybrid type structures[34, 40]. Previous studies revealed that apoB-100 glycan modifications can induce atherogenic properties on LDL[40]. In our study, the abundant mannose and complex/hybrid N-glycans were detected in hypercholesterolemic patients and rabbits compared with their corresponding controls. Meanwhile, severe atherosclerotic plaque was also exhibited in the cholesterol-fed rabbits (data are not shown). N-glycan structure analyses revealed that the core-fucosylated bi-antennary is the common major structure at all glycosylation sites[32]. We also found that several of the fucosylated, or fucosylated and sialylated modified N-glycans in hypercholesterolemic patients showed much higher level than healthy controls. These results suggested that the abundance of N-glycans in plasma may be associated with glycosylation modifications of several lipoproteins, such as apoB-100 or other plasma secreted proteins. It remains to be verified, however, what the pathophysiological significance of high glycan abundance in hypercholesterolemia is in future.

In conclusion, we have shown for the first time that plasma N-glycans are associated with hypercholesterolemia. Although the molecular mechanisms and pathophysiological significance remain unclear, these results provide an important clue that plasma N-glycans can become a new target for diagnosis of hypercholesterolemia in future.

## Supporting Information

S1 Fig MS/MS data for the N-glycans released from human plasma. Glycan compositions and sequences were assigned manually based on MS/MS analysis. Four representatives were used to show how to ascertain N-glycan structures. 1743.42 (high mannose) (A), 1501.25 (complex) (B), 1485.33 (fucosylated) (C) and 1930.33 (sialylated) (D). Structural formulas: blue square, N-acetylglucosamine; green circle, mannose; yellow circle, galactose; red triangle, fucose; purple diamond, N-acetylneuraminic acid.
(TIF)

S2 Fig The plasma N-glycan profiling from healthy subjects and hypercholesterolemic patients. N-glycan levels of two groups were presented by a heat map (A). Red color represents higher levels of N-glycans while blue color displays lower levels of N-glycans in plasma of hypercholesterolemic patients and healthy subjects. Peak intensity ratio of N-glycans to β-cyclodextrin was calculated to compare high-mannose (B), complex (undercorated) (C), complex (fucosylated) (D), complex (fucosylated and sialylated) (E), complex (sialylated) (F) and hybrid (G) from control and hypercholesterolemic patients. Data are expressed as the mean ± SEM. n = 9 for each group. *P<0.05, **P<0.01, ***P<0.001. HC, hypercholesterolemia; und, undercorated; fuc, fucosylated; sia, sialylated.
(TIF)

S3 Fig MS/MS data for the N-glycans released from rabbit plasma. Glycan compositions and sequences were assigned manually based on MS/MS analysis. Four representatives were used to show how to ascertain N-glycan structures. 1905.42 (high mannose) (A), 1542.17 (complex) (B), 1647.33 (fucosylated) (C) and 1769.25 (sialylated) (D). Structural formulas: blue square, N-acetylglucosamine; green circle, mannose; yellow circle, galactose; red triangle, fucose; purple diamond, N-acetylneuraminic acid.
(TIF)

S4 Fig The plasma N-glycan profiling from controls and hypercholesterolemic rabbits. N-glycan levels of two groups were indicated by a heat map (**A**). The intensity of red color represents higher levels of N-glycans while blue color represents lower levels of N-glycans. Peak intensity ratio of N-glycans to β-cyclodextrin was used to calculate high-mannose (B), complex (sialylated) (C) and complex (undercorated) (D) from control and hypercholesterolemic group. Data are expressed as the mean ± SEM. n = 5 for each group. *P<0.05, **P<0.01 vs. control. HCD, high cholesterol diet; und, undercorated; sia, sialylated.
(TIF)
