# Supplementary figures and images for: Plasma High-Mannose and Complex/Hybrid N-Glycans Are Associated with Hypercholesterolemia in Humans and Rabbits
Source: PLoS One. 2016 Mar 21;11(3):e0146982. doi: 10.1371/journal.pone.0146982 (PMC4801423; doi:10.1371/journal.pone.0146982)

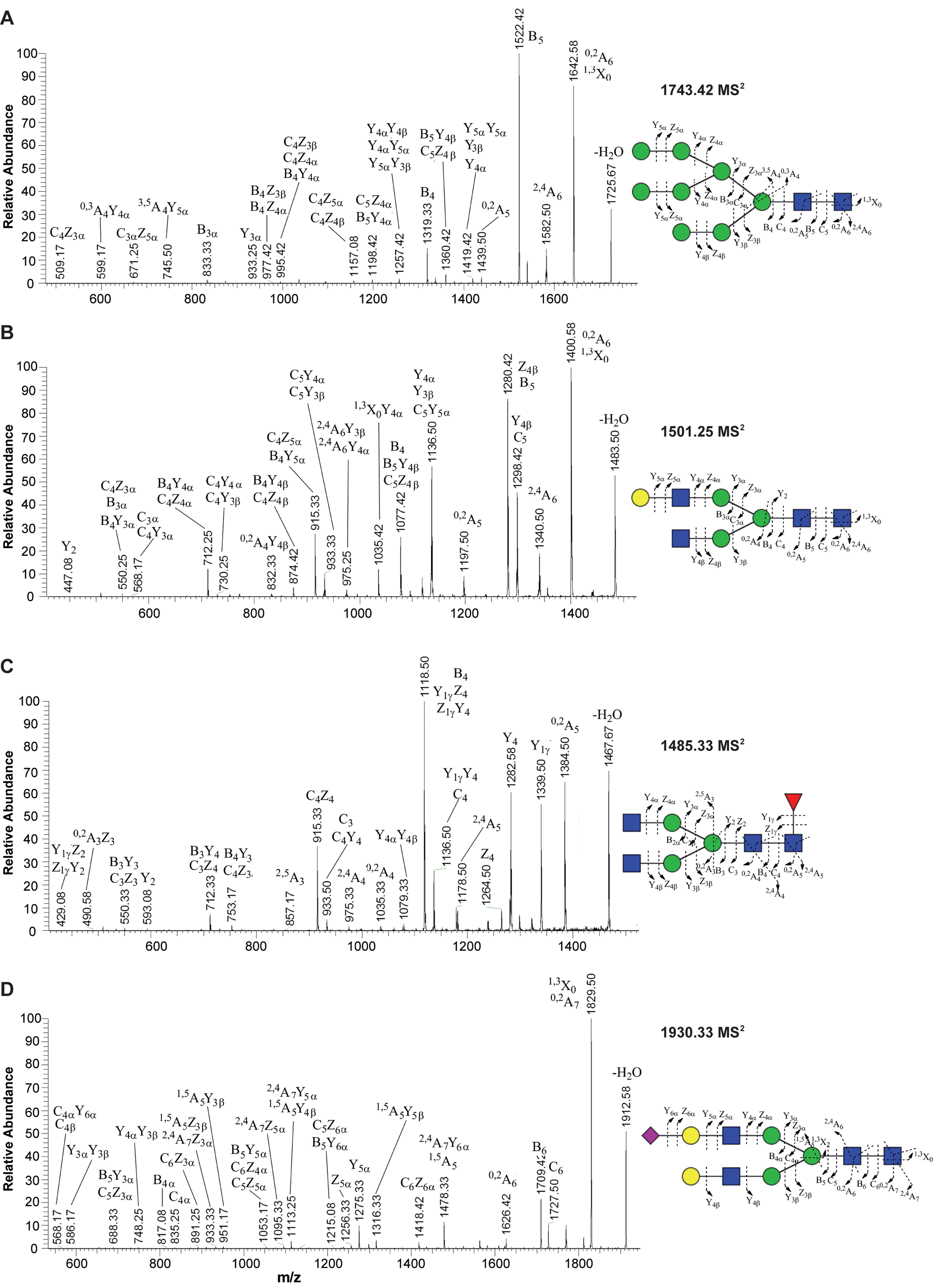

Supplement: S1 Fig — Glycan compositions and sequences were assigned manually based on MS/MS analysis. Four representatives were used to show how to ascertain N-glycan structures. 1743.42 (high mannose) (A), 1501.25 (complex) (B), 1485.33 (fucosylated) (C) and 1930.33 (sialylated) (D). Structural formulas: blue square, N-acetylglucosamine; green circle, mannose; yellow circle, galactose; red triangle, fucose; purple diamond, N-acetylneuraminic acid. (TIF) [file pone.0146982.s001.tif]

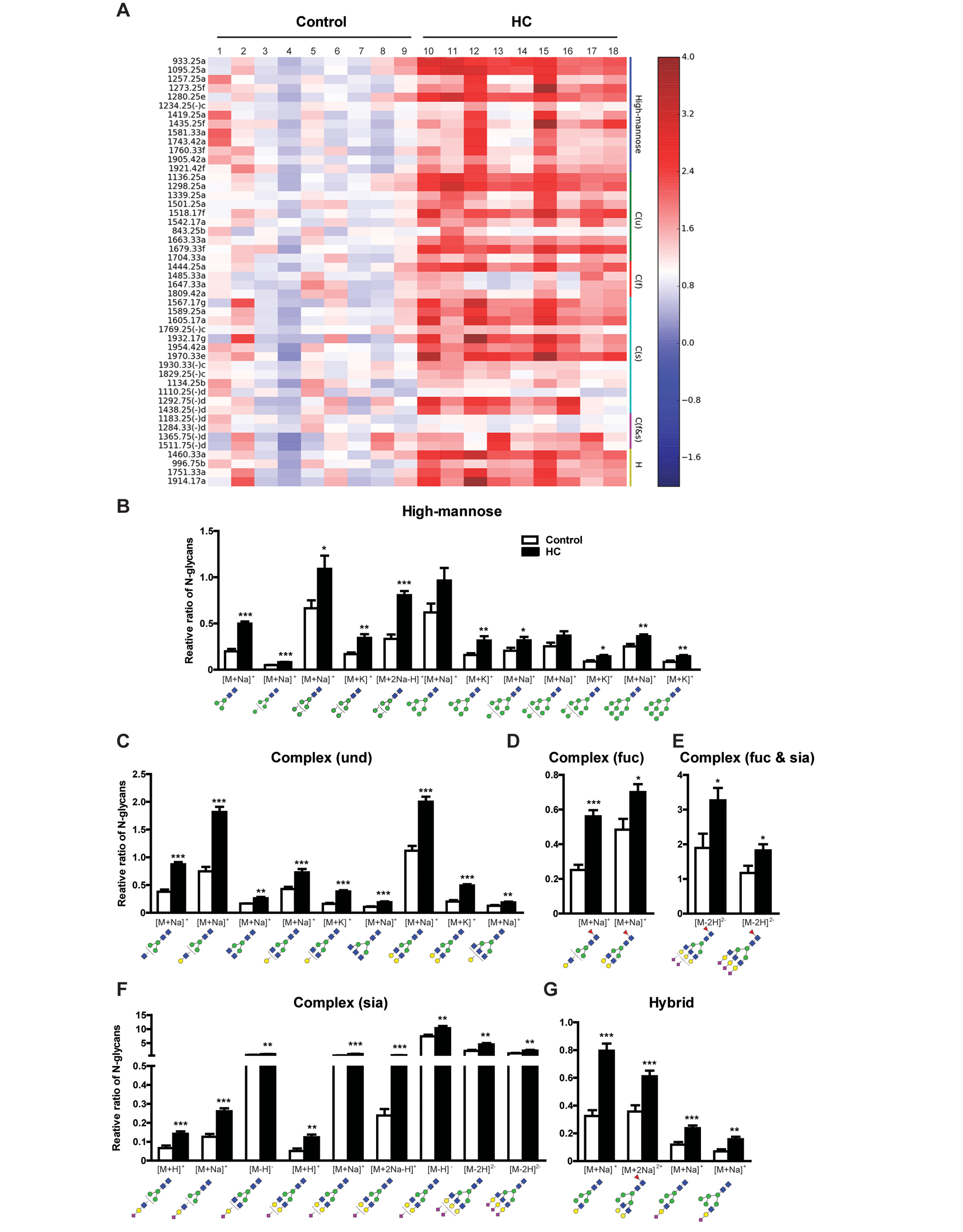

Supplement: S2 Fig — N-glycan levels of two groups were presented by a heat map (A). Red color represents higher levels of N-glycans while blue color displays lower levels of N-glycans in plasma of hypercholesterolemic patients and healthy subjects. Peak intensity ratio of N-glycans to β-cyclodextrin was calculated to compare high-mannose (B), complex (undercorated) (C), complex (fucosylated) (D), complex (fucosylated and sialylated) (E), complex (sialylated) (F) and hybrid (G) from control and hypercholesterolemic patients. Data are expressed as the mean ± SEM. n = 9 for each group. *P<0.05, **P<0.01, ***P<0.001. HC, hypercholesterolemia; und, undercorated; fuc, fucosylated; sia, sialylated. (TIF) [file pone.0146982.s002.tif]

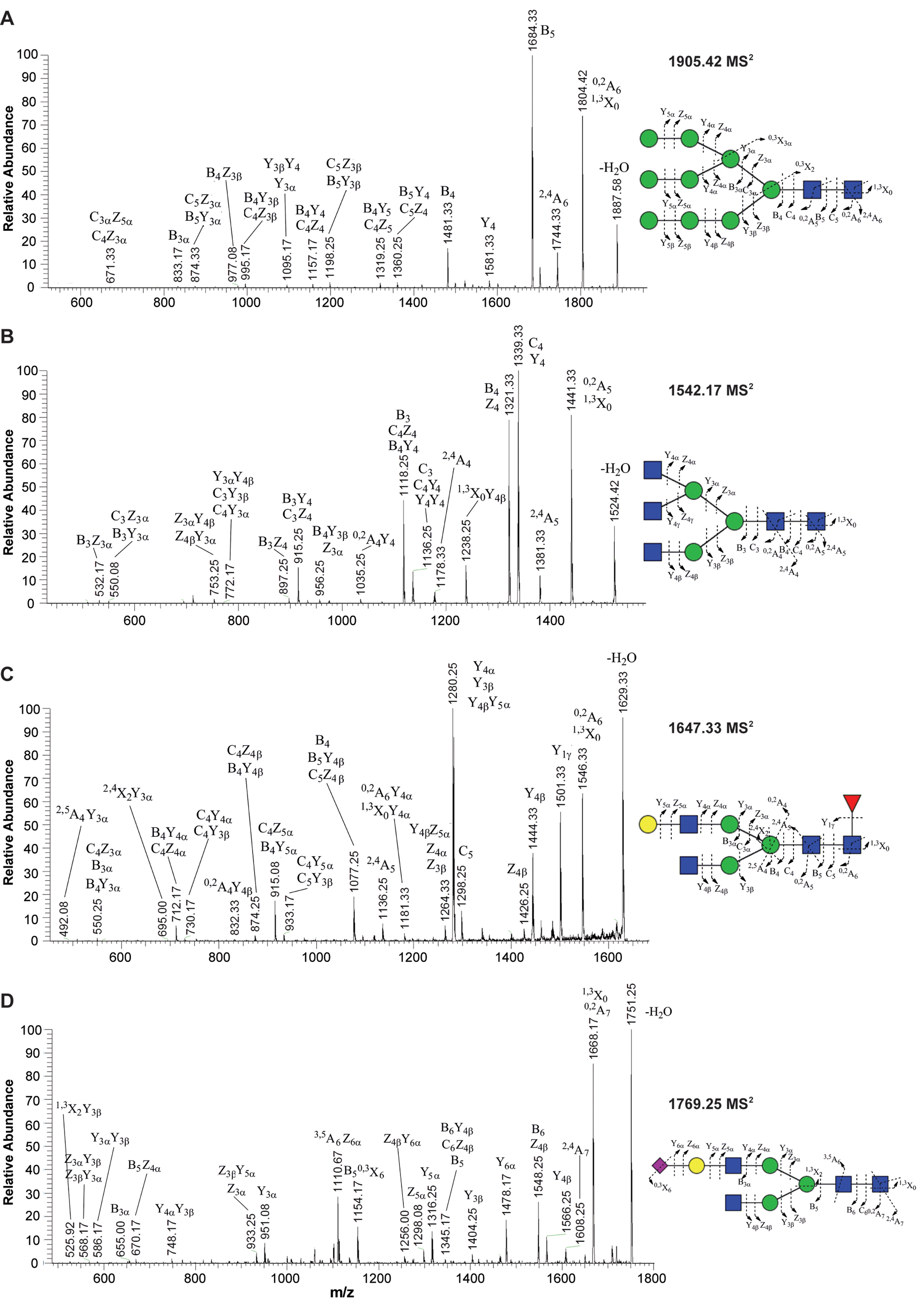

Supplement: S3 Fig — Glycan compositions and sequences were assigned manually based on MS/MS analysis. Four representatives were used to show how to ascertain N-glycan structures. 1905.42 (high mannose) (A), 1542.17 (complex) (B), 1647.33 (fucosylated) (C) and 1769.25 (sialylated) (D). Structural formulas: blue square, N-acetylglucosamine; green circle, mannose; yellow circle, galactose; red triangle, fucose; purple diamond, N-acetylneuraminic acid. (TIF) [file pone.0146982.s003.tif]

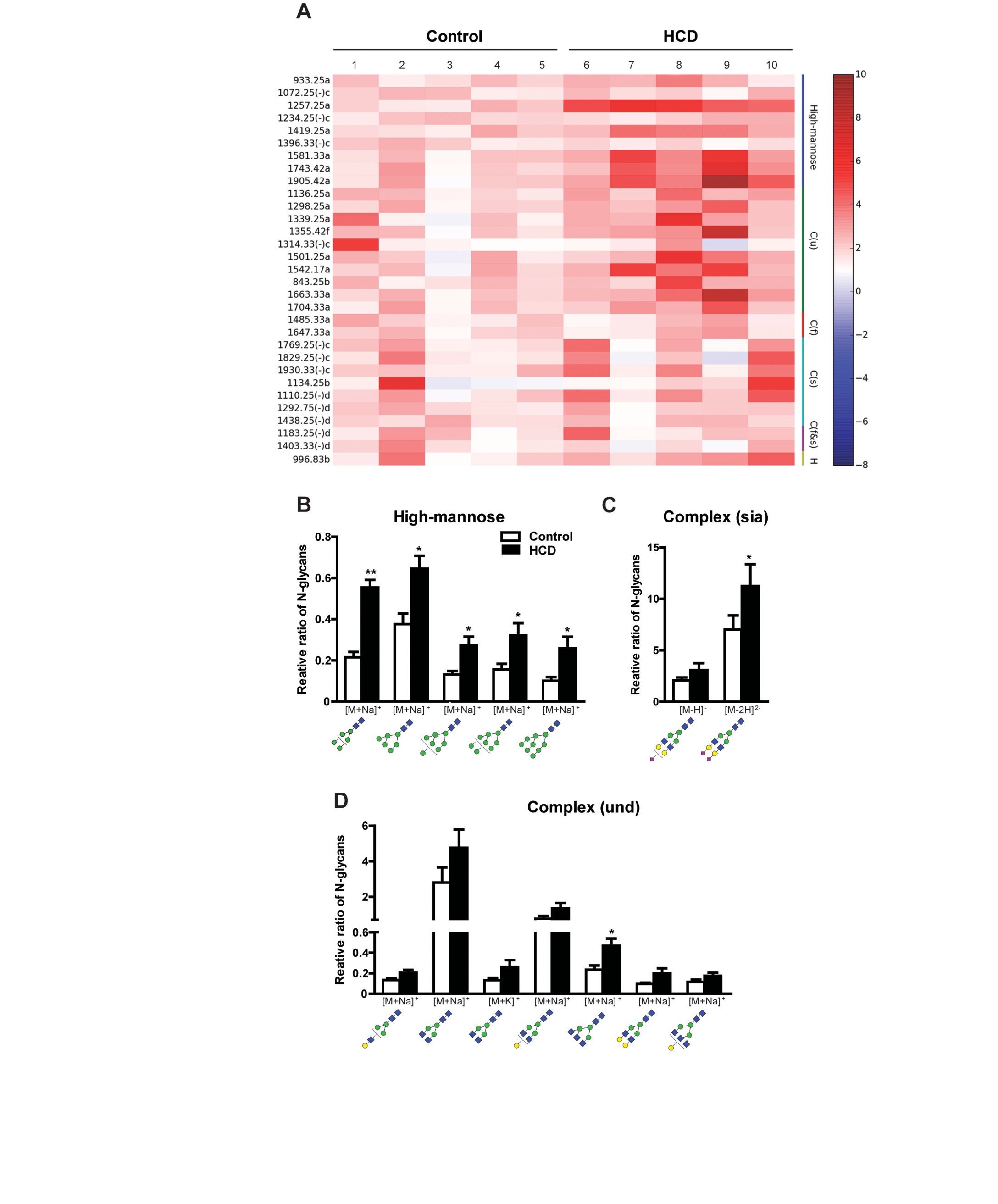

Supplement: S4 Fig — N-glycan levels of two groups were indicated by a heat map (A). The intensity of red color represents higher levels of N-glycans while blue color represents lower levels of N-glycans. Peak intensity ratio of N-glycans to β-cyclodextrin was used to calculate high-mannose (B), complex (sialylated) (C) and complex (undercorated) (D) from control and hypercholesterolemic group. Data are expressed as the mean ± SEM. n = 5 for each group. *P<0.05, **P<0.01 vs. control. HCD, high cholesterol diet; und, undercorated; sia, sialylated. (TIF) [file pone.0146982.s004.tif]
